# The relevance of ototoxicity induced by radiotherapy

**DOI:** 10.1186/s13014-023-02268-7

**Published:** 2023-06-03

**Authors:** Yan Huang, Hong Zhou, Fenglan An, Aimei Zhao, Jian Wu, Meihua Wang, Judong Luo

**Affiliations:** 1 Department of Radiotherapy, The Affiliated Changzhou Second People’s Hospital of Nanjing Medical University, Changzhou Medical Center, Nanjing Medical University, Changzhou, China; 2 Department of Head and Neck Surgery, Graduate School of Dalian Medical University, Dalian, China; 3 Department of Otolaryngology, Shidong Hospital, Yangpu District, Shidong Hospital Affiliated to University of Shanghai for Science and Technology, Shanghai, China; 4 The Third Department of Internal Medicine, Hospital of Traditional Chinese Medicine, Lingcheng, Dezhou, Shandong Province China; 5 Department of Obstetrics and Gynecology, Dongchangfu Maternal and Child Health Hospital of Liaocheng, Liaocheng, China; 6 Department of Head and Neck Surgery, The Affiliated Changzhou No.2 People’s Hospital of Nanjing Medical University, Changzhou, China; 7 Department of Pathology, Changzhou Tumor Hospital, Changzhou, China

**Keywords:** Radiation exposure, Radiotherapy, Radiation dose, Ototoxicity, Cochlea

## Abstract

**Background:**

The risk of ototoxicity, characterized by hearing impairment, tinnitus, or middle ear inflammation, is elevated in both child and adult cancer survivors who have undergone head-neck or brain radiation, or a combination of the two. To provide optimal care for these cancer survivors and minimize subsequent complications, it is crucial to comprehend the relationship between radiotherapy and ototoxicity.

**Methods:**

A comprehensive search of databases, including the Cochrane Library, PubMed, Embase, and Web of Science, was conducted from the inception of the knowledge base up until January 2023. The metafor-package was employed to compare ototoxicity rates in individuals receiving radiotherapy. Two independent assessors extracted data and analyzed targets using a random-effects model.

**Results:**

Out of the 28 randomized controlled trials (RCTs) included in the analysis, 25 were prospective RCTs. Subgroup analysis revealed that mean cochlear radiation dose, primary tumor location, radiotherapy modality, and patient age significantly influenced total hearing impairment. Intensity-modulated radiotherapy was associated with less ototoxicity than 2D conventional radiotherapy (OR, 0.53; 95% CI, 0.47–0.60; P = 0.73; I^2^ = 0%). Stereotactic radiotherapy appeared to be a superior option for hearing preservation compared to radiosurgery (OR, 1.44; 95% CI, 1.00–2.07; P = 0.69; I^2^ = 0%). Children demonstrated a higher risk of hearing impairment than adults. More than 50% of patients with vestibular neuroadenoma experienced hearing impairment following radiation therapy. A strong association was observed between the average cochlear radiation dose and hearing impairment. Increased cochlear radiation doses may result in a heightened risk of hearing impairment.

**Conclusion:**

Several risk factors for radiation-induced hearing impairment were identified in this study. High cochlear radiation doses were found to exacerbate the risk of hearing impairment resulting from radiation therapy.

**Supplementary Information:**

The online version contains supplementary material available at 10.1186/s13014-023-02268-7.

## Introduction

Ototoxicity, which can manifest as hearing impairment, tinnitus, and/or vertigo, is a recognized adverse effect associated with a group of antitumor therapies, including platinum chemotherapy, radiotherapy, or surgery involving the ear and auditory nerves [1]. Hearing impairment can lead to communication and social difficulties, ultimately reducing the quality of life. In children, hearing impairment can severely impair cognitive development as well as language and social skills. Although the structure of the human ear is formed at birth, the maturation of neural pathways and auditory structures continues during infancy and early childhood, making young children particularly vulnerable to radiotherapy-induced ototoxicity [2].

Radiation therapy (RT), which can be utilized as a single treatment modality or as an adjuvant treatment before and after surgery, is commonly used to treat patients with a variety of cancers. Patients with locally advanced and inoperable head and neck tumors are usually treated with cisplatin-based chemoradiotherapy. Cisplatin or carboplatin-based chemotherapy drugs are known to cause ototoxicity [3]. Winther et al. discovered that inner ear radiation in guinea pigs resulted in extensive degeneration of hair cells outside Corti organs. Concurrently, radiation therapy to the temporal bone led to Corti organ damage and auditory vestibular nerve atrophy [4]. In radiation therapy for head, neck, or brain malignancies, the middle ear, inner ear, and brainstem may be exposed to high doses of ionizing radiation [5]. The underlying physiological processes leading to hearing impairment may vary depending on the location of radiation-induced lesions. If hearing impairment arises from damage to middle ear components, such as eustachian tubes or ossicles, it is classified as conductive. In contrast, sensorineural hearing loss (SNHL) results from lesions in the cochlea or the auditory system’s posterior section [6].

Despite the prevalence and severity of ototoxicity following radiotherapy, it has seldom been reported in radiation oncology literature. The correlation between cochlear radiation dose and subsequent morbidity has rarely been documented. The objective of this study is to assess the incidence of various factors that may contribute to radiotherapy-induced ototoxicity.

## Methods

This meta-analysis was conducted in accordance with the Preferred Reporting Items for Systematic Reviews and Meta-Analyses (PRISMA) [7].

### Data sources and searches

Databases, including the Cochrane Library, PubMed, Embase, and Web of Science, were searched from inception until January 2023. Medical Subject Headings (MeSH) and text word combinations were employed to create three subsets of references: the first subset encompassed radiotherapy (such as intensity-modulated radiotherapy, proton radiotherapy, carbon ion radiotherapy, photon radiation, gamma knife, stereotactic radiotherapy, etc.); the second subset involved complications related to hearing (namely, ototoxicity, hearing impairment, and hearing loss); and the third subset pertained to cancer. After an initial screening of titles or abstracts, two independent reviewers (YH, HZ) assessed the full text of relevant publications and the reference lists for final inclusion. Additionally, references considered potentially relevant were searched and thoroughly evaluated.

### Study selection

Studies were included based on the following criteria: (1) studies that reported hearing impairment in cancer patients due to RT as a first-line treatment; (2) hearing outcomes obtained from pure tone audiograms (either air and bone conduction or bone conduction alone) conducted before and after treatment; (3) studies providing the number of individuals evaluable for toxicity following radiotherapy and the number of individuals with hearing impairment; (4) studies that clearly defined hearing impairment and offered sufficient irradiation information to quantify the effect; and (5) studies that were randomized controlled trials, excluding one-arm trials. All criteria needed to be met for study inclusion. Exclusion criteria encompassed postoperative studies, single-arm studies, case reports, reviews, meeting minutes or abstracts, articles not published in English, and studies with cisplatin as monotherapy.

### Data extraction

Two evaluators independently employed standardized forms to extract and summarize the following data: first author, year of publication, study ID, country, cancer type, radiotherapy design, radiotherapy mode, cochlear radiation dose, total number of patients, number of patients for safety analysis, standard version of general terms for adverse events, rate of hearing impairment, and frequency of tinnitus and vertigo symptoms. The standard for general terms of adverse events served as the most commonly used tool to evaluate the type and severity of adverse events in clinical practice, featuring a grading scale and clear definitions.

### Quality assessment

Two reviewers (AZ, JW) evaluated the risk of bias based on the original studies, utilizing the Cochrane Collaboration’s tool. Five aspects of adequacy were assessed: random sequence generation, allocation concealment, blinding, outcome assessment, and outcome reporting [8]. Each item was assigned an assessment indicator related to risk of bias, classified as yes, no, or unclear. Any disagreements regarding study selection, data extraction, and quality assessment were resolved through discussions with statistical experts [9].

### Data synthesis and statistical analysis

Meta-analysis was performed using R(4.2.1) statistical software (metafor and meta package). Fixed-effect or random-effects models were employed to estimate event rates and their corresponding 95% confidence intervals. Forest plots were constructed to summarize data and incidence for each analysis group. The Cochran Q statistic and the I^2^ statistic were utilized to assess statistical heterogeneity [10]. When I^2^ exceeded 25%, 50%, or 75%, it indicated low, medium, or high heterogeneity, respectively. If significant heterogeneity was present, a random-effects model was used. A simple analysis of funnel plots offered a useful test for possible bias in meta-analyses [11]. Otherwise, a fixed-effects model was applied. Meta-Analyst was used to generate pooled rates of different ototoxic events for treatment. Subgroup analysis was conducted according to median cochlear radiation dose. Finally, sensitivity analysis was performed to evaluate the stability of the results.

## Results

### Systematic review and characteristics

**Fig. 1:**
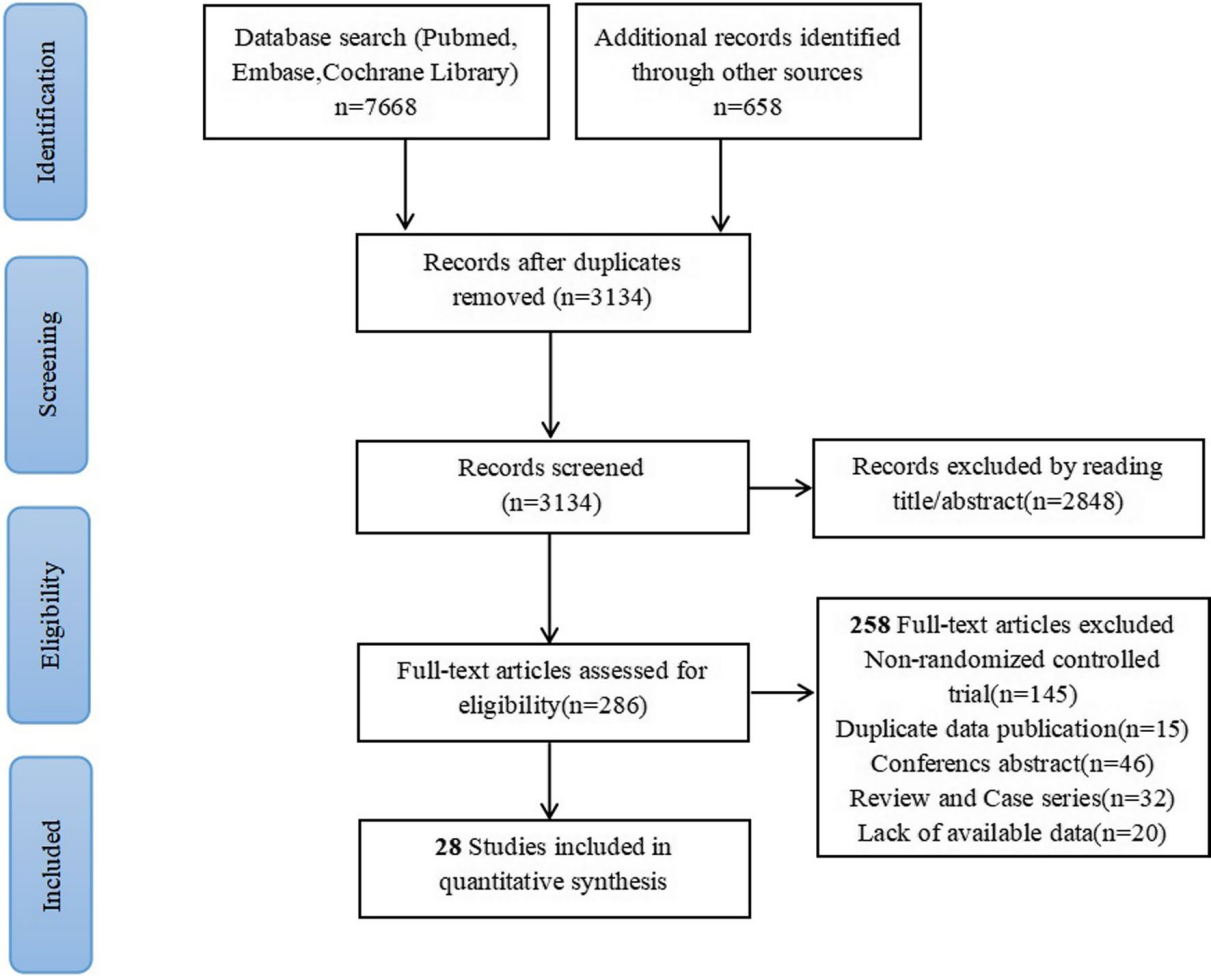
Flow chart of document screening

After 5192 duplicates were deleted and filtered by title and abstract, 286 of the 3134 records initially searched were reviewed in full. Due to insufficient data or lack of full text in meta-analysis, we excluded 20 studies. Finally, 28 eligible studies were included, including 25 prospective randomized controlled trial and 3 retrospective randomized controlled trials [12–14]. The radiotherapy modes included IMRT (intensity-modulated radiotherapy), SRT (Stereotactic radiotherapy), 3D-CRT (three-dimensional conformal RT), Conventional-RT, Proton-RT, Radiosurgery, HFRT (hyperfractionated RT), ART (accelerated RT). The median age ranged from 3 to 87. Patients under 18 years old participated in 3 studies, and the median age of the adult groups was greater than 18 years old. Twelve countries were included in the study: China (n = 9), U.S.A (n = 7), UK (n = 2), Sweden (n = 2), Canada (n = 1), Germany (n = 1), Japan (n = 1), Thailand (n = 1), Norway (n = 1), Singapore (n = 1), Spain (n = 1), Netherlands (n = 1). CTCAE (Common Terminology Criteria for Adverse Events) grading system was used to define ototoxic effects in 15 studies (i.e., Grade 1: Threshold shift of 15–25 dB averaged at two contiguous frequencies; Grade 2: Threshold shift of > 25 dB averaged at two contiguous frequencies; Grade 3: Threshold shift of > 25 dB averaged at three contiguous frequencies; Grade 4: > 80 dB at 2 kHz and above). Two study used Brock criteria to evaluate ototoxicity (i.e., Grade 0 to 1: < 40 dB on all frequencies or ≥ 40 dB at 8 kHz; Grade 2: ≥ 40 dB at 4 kHz; Grade 3 to 4: ≥ 40 dB at 2-1 kHz). Two studies used Gardner Robertson scale to judge ototoxicity. One study used the Pediatric Oncology Group (POG) to define the effects of ototoxicity. More information about the included study population and programs has been listed in Table 1.The detailed process of retrieval was shown in Fig. 1.

**Table 1:** Characteristics of included documents

First author	Year	Country	Trial type	Number trial name	Primary site,No. (%)	T stage (%)	Pathologic stage (%)	Radiotherapy dose	Control arm treatment	Patients in control arm (n)	Age, median (IQR)	ECOG status, No. (%)	Experimental arm treatment	Patients in experimental arm (n)	Age
Kiyota [15]	2022	Japan	Prospective, Phase III Trial	JCOG1008	Oral cavity 121(46) Larynx 23(9) Oropharynx 35(14) Hypopharynx 82(31)	T1 + T2 (33) T3 + T4 (67)	III (8) IVA (88) IVB (3)	3D-CRT OR IMRT: 66 Gy in 33 fractions over 6.5 weeks	3-weekly Cisplatin + RT	132	62(55–68)	= 0 92(70) = 1 40(30)	Weekly Cisplatin + RT	129	61(53–66)
You [16]	2020	China	Prospective, Phase III Trial	NCT02111460	Nasopharyngeal Carcinoma	T1 + T2 (11.1) T3 + T4 (88.9)	NA	IMRT: Prescribed doses were 70 Gy to GTVnx, 5 times per week	Chemo	63	47(39–52)	NA	Chemo + RT	63	46 (37–52)
Nichols [17]	2019	Canada	Prospective, Phase II Trial	ORATOR	Oropharyngeal carcinoma	T1 (44) T2 (56)	NA	IMRT: 70 Gy in 35 fractions over 7 weeks	TORS + ND	34	58.1 (52.6–64.5)	= 0 30(88) = 1 4 (12)	IMRT	34	60.0 (53.2–65.2)
Brown [18]	2017	USA	Prospective, Phase III Trial	NCCTG N107C/CEC·3	metastatic brain disease	NA	NA	SRS: 12–20 Gy fraction with dose by surgical cavity volume WBRT: 30 Gy in ten fractions	WBRT	96	62 (54–68)	= 0 33 (34) = 1 56 (58) = 2 7 (7)	SRS	98	61 (54–66)
Wong Kein Low [19]	2006	Singapore	Prospective Trial	NA	Nasopharyngeal Carcinoma	NA	NA	Conventional-RT: a dose of 70 Gy in 35 fractions, 2 Gy per fraction	Chemoradiotherapy	51	47(15–74)	NA	Radiotherapy	44	43(30–70)
Breivik [20]	2013	Norway	Prospective, Phase II Trial	NA	Vestibular schwannoma	NA	NA	GKRS: dose was 12 Gy to the periphery of the tumor with minimum 95% coverage	Conservative management	124	55.7	NA	GKRS	113	57.7
Nutting [21]	2018	United Kingdom	Prospective, Phase III Trial	COSTAR; CRUK/08/004	Parotid cancer	T1 + T2 (62.7) T3 + T4 (32.7)	NA	3D-CRT or CS-IMRT dose of 60 Gy or 65 Gy in 30 daily fractions	3D-CRT	54	59 (19–88)	NA	CS-IMRT	56	57 (20–87)
Lee [12]	2009	China	Retrospective study	NA	Nasopharyngeal Carcinoma	T1 + T2 (40) T3 + T4 (60)	I-IIB	(15) III-IVB (85)	3D-CRT 2-Gy daily fractions to a total dose of 70 Gy	Chemo + 3D-CRT	196	48(17–76)	NA	3D-CRT	171
Buckner [22]	2006	USA	Prospective, Phase III Trial	NA	Glioblastoma multiforme	NA	NA	Conventional RT: 1.80 Gy for 36 days (64.8 Gy) ART: 1.60 Gy twice daily for 15 days (48.0 Gy)	Chemo + ART	103	55	= 0 40(38.8) = 1 48 (46.6) = 2 15 (14.6)	Chemo + Conventional-RT	98	56
Mandell [23]	1999	USA	Prospective, Phase III Trial	POG-9239	Brainstem tumor	NA	NA	Conventional-RT:180 cGy/d to 5400 cGy HFRT:117 cGy/d to 7020 cGy	HFRT	64	3–21	NA	Conventional-RT	66	3–21
Wai-Tong Ng [13]	2018	China	Retrospective study	NPC-9901 NPC-9902	Nasopharyngeal Carcinoma	NA	III-IVB(100)	3DCRT: 2 Gy per fraction, 5 fractions per week. total dose of 66 Gy	Chemo + 3D-CRT	223	> 70	≤ 2(100)	3D-CRT	218	> 70
Lertbutsayanukul [24]	2018	Thailand	Prospective, Phase III Trial	NA	Nasopharyngeal Carcinoma	T1 + T2 (60) T3 + T4 (40)	I–II(14.8) III–IVA (69.4) IVB (15.7)	SEQ-IMRT:2 Gy × 25 fractions to low-risk target SIB-IMRT:doses of 56 and 70 Gy in 33 fractions	SIB-IMRT	107	50.4	NA	SEQ-IMRT	102	48.3
Lannering [25]	2012	Sweden	Prospective, Phase III Trial	HIT-SIOP PNET 4	Medulloblastoma	NA	NA	Conventional-RT:42 days in 30 fractions of 1.8 Gy; HFRT:68 fractions,1.0 Gy twice per day	HFRT + Chemo	68	4–21	NA	Conventional-RT + Chemo	78	4–21
Xiao-Yun Li [26]	2022	China	Prospective, Phase II Trial	NCT02871518	Nasopharyngeal Carcinoma	T1 + T2 (12.4) T3 + T4 (87.6)	III(79.8) IVa(8.7) IVb(11.4)	IMRT:target volume (PTV)nx, PTVnd, PTV1, and PTV2 were 68–70 Gy, 64–70 Gy, 60–64 Gy, and 50–54 Gy	Three-Cycle cisplatin + RT	166	46 (38–53)	NA	Two-Cycle cisplatin + RT	166	48(38–53)
Kessel [27]	2017	Germany	Prospective, Phase II Trial	NA	Vestibular schwannoma	NA	NA	RS:median dose of 12 Gy SRT:median dose of 54 Gy and a median single dose of 1.8 Gy	RS	56	63(16–85)	NA	SRT	128	59(17–82)
Paulino [14]	2018	USA	Retrospective study	NA	Medulloblastoma	NA	NA	Standard-risk patients received 18–23.4 Gy/CGE high-risk patients received 36–39.6 Gy/CGE	Proton-RT	38	7.6 (2.9–14.5)	NA	IMRT	46	9.0 (3.0–18.0)
Tang [28]	2018	China	Prospective, Phase III Trial	NCT01540136	Nasopharyngeal Carcinoma	T1 + T2 (23.5) T3 + T4 (76)	II (12) III (67) IVA(14.5) IVB(5.5)	2.0–2.33 Gy per fraction with five fractions per week for 6–7 weeks	Nedaplatin + RT	201	44 (18–65)	90–100 (96) 70–80 (4)	Cisplatin + RT	201	45 (20–64)
Marshall [29]	2005	USA	Prospective, Phase II Trial	NA	Glioblastoma	NA	NA	Conventional-RT: 180 cGy once a day for 36 days ART:160 cGy twice daily for 15 days	Chemo + ART	113	52.8 ± 11.8	NA	Chemo + Conventional-RT	117	55.8 ± 11.5
Poon [30]	2021	China	Prospective Trial	NCT02339701	Nasopharyngeal Carcinoma	T1 + T2 (100)	I (47.7) II (52.3)	IMRT: 66/60 Gy in 33 fractions Conventional-RT:40 Gy in 20 fractions/4 weeks	IMRT	28	48 (29–64)	= 0 8(80) = 1 2 (20)	Conventional-RT	28	43 (30–59)
You-Ping Liu [31]	2021	China	Prospective, Phase III Trial	ChiCTR-TRC-11001573	Nasopharyngeal carcinoma	T1 + T2 (32) T3 + T4 (48)	II(21.5) III-IV(58.5)	IMRT: 60–70 Gy in 27–35 fractions	Endoscopic nasopharyngectomy	100	46 (38–55)	90–100(95) 70–80(5)	IMRT	100	49 (41–54)
Hisham Mehanna [32]	2019	United Kingdom	Prospective, Phase III Trial	ISRCTN33522080	Oropharyngeal cancer	T1 + T2 (65) T3 + T4 (35)	NA	IMRT: a dose of 65 Gy or more	Cetuximab + IMRT	168	57(51–64)	= 0 149(91) = 1 15(9)	Cisplatin + IMRT	166	56.5 (52–62)
Gillison [33]	2019	USA	Prospective, Phase III Trial	RTOG 1016	Oropharyngeal cancer	T1 + T2 (62) T3 + T4 (38)	III (7) IV (93)	IMRT: 70 Gy in 35 fractions over 6 weeks at six fractions per week	Cetuximab + IMRT	399	58 (52–63)	= 0 300 (75) = 1 99 (25)	Cisplatin + IMRT	406	58 (52–63)
Maria Gebre-Medhin [34]	2021	Sweden	Prospective, Phase III Trial	ARTSCAN III	Head and neck squamous cell carcinomas	T1 + T2 (52.5) T3 + T4 (47)	III (10.5) IV (89.5)	IMRT: 68.0 Gy to the primary tumor and lymph node metastases	Cetuximab + IMRT	146	61(33–77)	= 0 132(90) ≥ 1 14(10)	Cisplatin + IMRT	145	61(45–75)
Hitt [35]	2022	Spain	Prospective, Phase III Trial	NA	Head and neck cancer	T1-3(38.3) T4(61.1)	III (7.3) IVA(72.7) IVB(19.6)	2 Gy per fraction for a total of 35 fractions	Cetuximab + Conventional-RT	202	58 (29–71)	= 0 141(70) = 1 61 (30)	Cisplatin + Conventional-RT	205	57 (40–73)
Peng [36]	2012	China	Prospective Trial	NA	Nasopharyngeal carcinoma	NA	I (4) II (28) III(48) IV(20)	IMRT optic chiasm was 54 Gy, 45 Gy for the spinal cord Conventional-RT 66 Gy with 2.0 Gy/fraction	IMRT	306	46.7 ± 12.5	= 0–1 288 ⩾218	Conventional-RT	310	44.8 ± 13.6
Fu [37]	2000	USA	Prospective Trial	RTOG 9003	Head and neck squamous cell carcinomas	T1 + T2 (33) T3 + T4 (67)	II (4) III(30) IV(66)	Conventional-RT:70 Gy/35 fractions/7 weeks ART:5 days/week to 81.6 Gy/68 fractions/7 weeks HFRT:67.2 Gy/42 fractions/6 weeks	ART,HFRT	263	60	60–80 (38) 90–100 (62)	Conventional-RT	268	60
Lee [38]	2015	China	Prospective Trial	NPC-0501	Nasopharyngeal carcinoma	T1 + T2 (21) T3 + T4 (79)	III (71) IVA-IVB (29)	66 Gy for T1-T2a tumors	ART	104	49	= 0 70 (67) = 1 33 (32) = 2 1 (1)	Conventional-RT	160	48
Meijer [39]	2003	Netherlands	Prospective Trial	NA	Vestibular schwannoma	NA	NA	2500 cGy was given(5*500 cGy)	RS	49	63	NA	SRT	80	43

ECOG status or Karnofsky score	Outcome measure	Mean Cochlear radiation dose	Outcome definition	Median follow-up
= 0 93(72) = 1 36(28)	Audiometry	NA	NA	26.4(IQR,14.2–42.7)mo
NA	Audiometry	NA	CTCAE(3.0)	26.7(IQR,17.2–33.5)mo
= 0 30(88) = 1 4 (12)	Audiogram	NA	CTCAE(4.0)	25(IQR, 20–33)mo
= 0 39 (40) = 1 49 (50) = 2 10 (10)	Audiometry	NA	CTCAE(4.0)	11.1(IQR, 5.1–18.0)mo
NA	Pure-tone audiograms	NA	NA	24mo
NA	Audiometry	NA	Gardner-Robertson scale (GR)	55(IQR,10–132)mo
NA	Audiometry	3D-CRT: 56.2 Gy CS-IMRT: 35.7 Gy	CTCAE(3.0)	49.9(IQR, 37.7–61.9)mo
52(20–84)	NA	Audiometry	RT:51 Gy Chemo + RT:56 Gy	CTCAE(3.0)
= 0 38(38.8) = 1 47(48.0) = 2 13(13.3)	Audiometry	NA	NA	NA
NA	Audiograms;BAER	NA	standard NCI common toxicity criteria	6-44mo
≤ 2(100)	Audiometry	NA	NA	NA
NA	Audiometry	SEQ-IMRT:45.14 ± 9.26 Gy SIB-IMRT:44.28 ± 8.60 Gy	CTCAE(4.03)	NA
NA	Audiometry	Conventional-RT:46.7 Gy HFRT:56.8 Gy	HIT;Brock	57.6 (range,1.2–99.6)mo
NA	Audiometry	NA	CTCAE(4.0)	37.7 (IQR, 33.0–46.1)mo
NA	Audiometry	NA	Gardner-Robertson Scale	90(range,0–172.8)mo
NA	Audiometry,ABR	IMRT: 37.5 ± 5.8 Gy Proton-RT:31.6 ± 8.5 Gy	Brock; CTCAE(3.0); POG; SIOP Boston	56 (range,13–101)mo
90–100(95) 70–80 (5)	Audiometry	NA	CTCAE(3.0)	48(IQR, 43–55)mo
NA	Audiometry	NA	NCI Common Toxicity Criteria grading (2.0)	NA
= 0 11(100)	Audiometry	NA	NA	186(IQR,180–189.6)mo
90–100(94) 70–80(6)	Audiometry	NA	CTCAE(3.0)	56.0(IQR, 42.0–69)
= 0 142(87) = 1 22(13)	Audiometry	NA	CTCAE(4.0)	25.9(IQR,25.5–26.0)
= 0 295 (73) = 1 111 (27)	Audiometry	NA	CTCAE(4.0)	54mo
= 0 133(92) ≥ 1 12(8)	Audiometry	NA	CTCAE(4.0)	39(IQR, 30–54)mo
= 0 155(76) = 1 50 (24)	Audiometry	NA	CTCAE(3.0)	43.9(IQR,64.4–21.2)mo
= 0–1 300 ⩾210	Audiometry	NA	CTCAE(3.0)	42(range,1–83)mo
60–80 (38) 90–100 (61)	Audiometry	NA	NA	23mo
= 0 116 (73) = 1 43 (27) = 2 1 (0.6)	Audiometry	NA	CTCAE(3.0)	39,6(range,1.2–85.2)mo
NA	Audiometry	NA	NA	33(range,12–107)mo

*IQR* interquartile range; *RT* radiotherapy; *Chemo* chemotherapy; *3D-CRT* three-dimensional conformal radiation therapy; *IMRT* intensity-modulated radiotherapy; TORS + ND = transoral robotic surgery and neck dissection; *ECOG* Eastern Cooperative Oncology Group; *WBRT* Whole brain radiotherapy; *SRT* stereotactic radiotherapy; *RS* radiosurgery; *GKRS* gamma knife radiosurgery; *CS-IMRT* cochlear-sparing IMRT; *ART* accelerated RT; *HFRT* hyperfractionated radiotherapy; *BAER* brain auditory evoked response; *SEQ-IMRT* Sequential-IMRT; *SIB-IMRT* simultaneous integrated boost-IMRT; *HIT* German Hirntumor study grading system; *SRT* stereotactic radiotherapy; *RS* radiosurgery; *ABR* auditory brainstem response; *POG* Pediatric Oncology Group; SIOP Boston = International Society of Pediatric Oncology Boston; *NCI* National Cancer Institute; *CTCAE* Common Terminology Criteria for Adverse Events;*mo* month

#### Risk of bias assessment

The risk of bias was assessed for each included study (Additional file 1: Fig. S1). The overall risk of bias was low. Two studies did not mention the randomization process [18, 26, 34], while the third study did not conceal selective reporting bias [34]. Another study did not report measurements or determinations of whether results differed between experimental groups [30]. Despite these inclusions, some concerns regarding the risk of bias remained.

### Ototoxicity

#### Hearing impairment

**Fig. 2:**
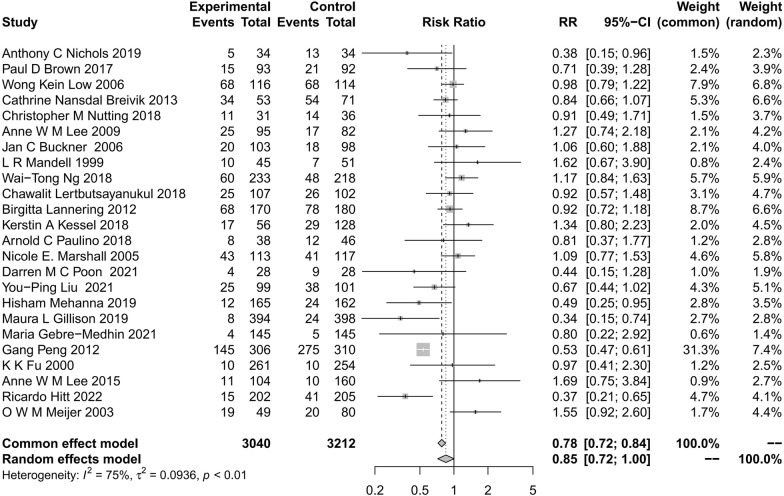
Summary total hearing impairment for all included studies

The 28 included studies compared the risk of all levels of hearing impairment effects in cancer patients receiving first-line therapy as radiation therapy (Fig. 2). The ratio between the experimental group and the control group under the random-effect model was 0.85 (95% CI, 0.71–1.00; P < 0.01; I^2^ = 75%). All RCTs were combined to compare the ratio of the experimental arm to the control arm, and the heterogeneity was high. Since the experimental design of each included RCT varied, subgroup analyses were performed based on population characteristics, original tumor, radiotherapy modality, and mean cochlear dose to explore potential sources of heterogeneity.

##### Subgroup analysis of the association of RT with hearing impairment by irradiation design mode

**Fig. 3:**
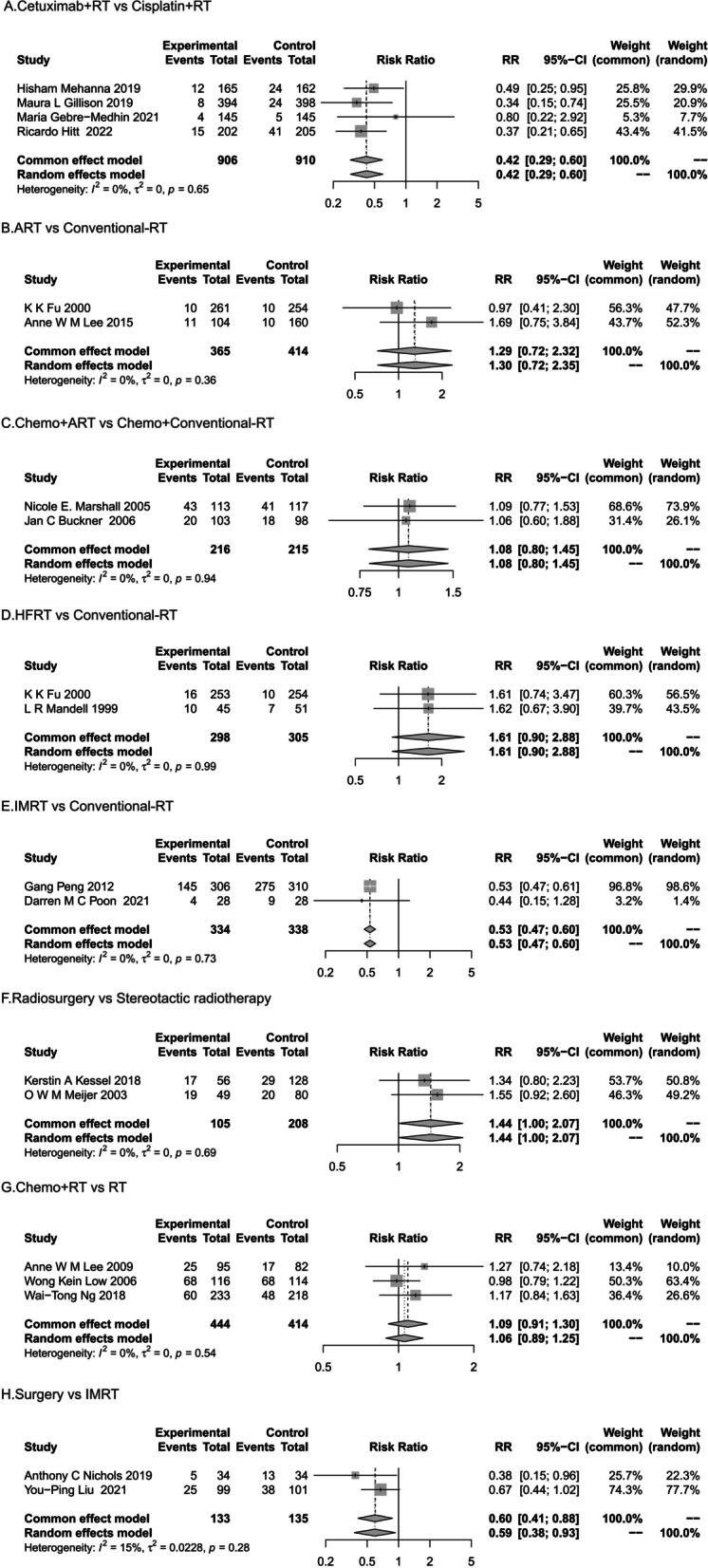
Subgroup Analysis of the Association of RT with Hearing impairment by Irradiation design mode

Four trials involved the combination of Cetuximab and RT compared to the combination of Cisplatin and RT (Fig. 3A). Three trials were designed with Cetuximab + IMRT versus Cisplatin + IMRT, and one trial with Cetuximab + Conventional-RT versus Cisplatin + Conventional-RT. The combined OR (Odds Ratio) value was 0.42 (95% CI, 0.29–0.6; P = 0.65; I^2^ = 0%). The result suggested that Cetuximab combined with radiotherapy resulted in lower hearing impairment than Cisplatin combined with RT.

Two trials compared ART with conventional-RT for ototoxicity (Fig. 3B). Pooled results suggest that hearing impairment after ART irradiation may be more severe than conventional radiotherapy (OR, 1.30, 95% CI, 0.72–2.35; P = 0.36; I^2^ = 0%). Two studies demonstrated the combination of ART and chemotherapy compared to conventional radiotherapy and chemotherapy (Fig. 3C). The results of the forest plot also showed that ART combined with chemotherapy is more ototoxic than conventional radiotherapy (OR, 1.08, 95% CI, 0.80–1.45; P = 0.94; I^2^ = 0%).

Two studies reported differences in ototoxicity between HFRT and conventional radiotherapy (Fig. 3D). Forest plot results showed that hearing impairment with HFRT was greater than loss with conventional radiotherapy (OR, 1.61, 95% CI, 0.9–2.88; P = 0.99; I^2^ = 0%). The advantages of IMRT can be seen in comparison with the hearing impaired population of Conventional-RT (OR, 0.53, 95% CI, 0.47–0.60; P = 0.73; I^2^ = 0%) (Fig. 3E).

Compared with SRT, RS irradiation caused more severe hearing damage (OR, 1.44, 95% CI, 1.00–2.07; P = 0.69; I^2^ = 0%) (Fig. 3F). Three trials covered chemotherapy combined with radiotherapy and radiotherapy alone. Two items were Chemotherapy + 3D-CRT compared with 3D-CRT, and one item was Chemotherapy + Conventional-RT compared with Conventional-RT. A summary analysis showed that chemotherapy combined with radiotherapy had a higher risk of ototoxicity than radiotherapy alone (OR, 1.06, 95% CI, 0.89–1.25; P = 0.54; I^2^ = 0%) (Fig. 3G).

In the comparison of surgery with radiotherapy, one trial involved transoral robotic surgery versus IMRT, while the other examined nasal endoscopic surgery versus IMRT. Surgical removal demonstrated higher hearing preservation than IMRT (OR, 0.59, 95% CI, 0.38–0.93; P = 0.28; I^2^ = 15%) (Fig. 3H).

##### Subgroup analysis of the association of RT with hearing impairment by age

**Fig. 4:**
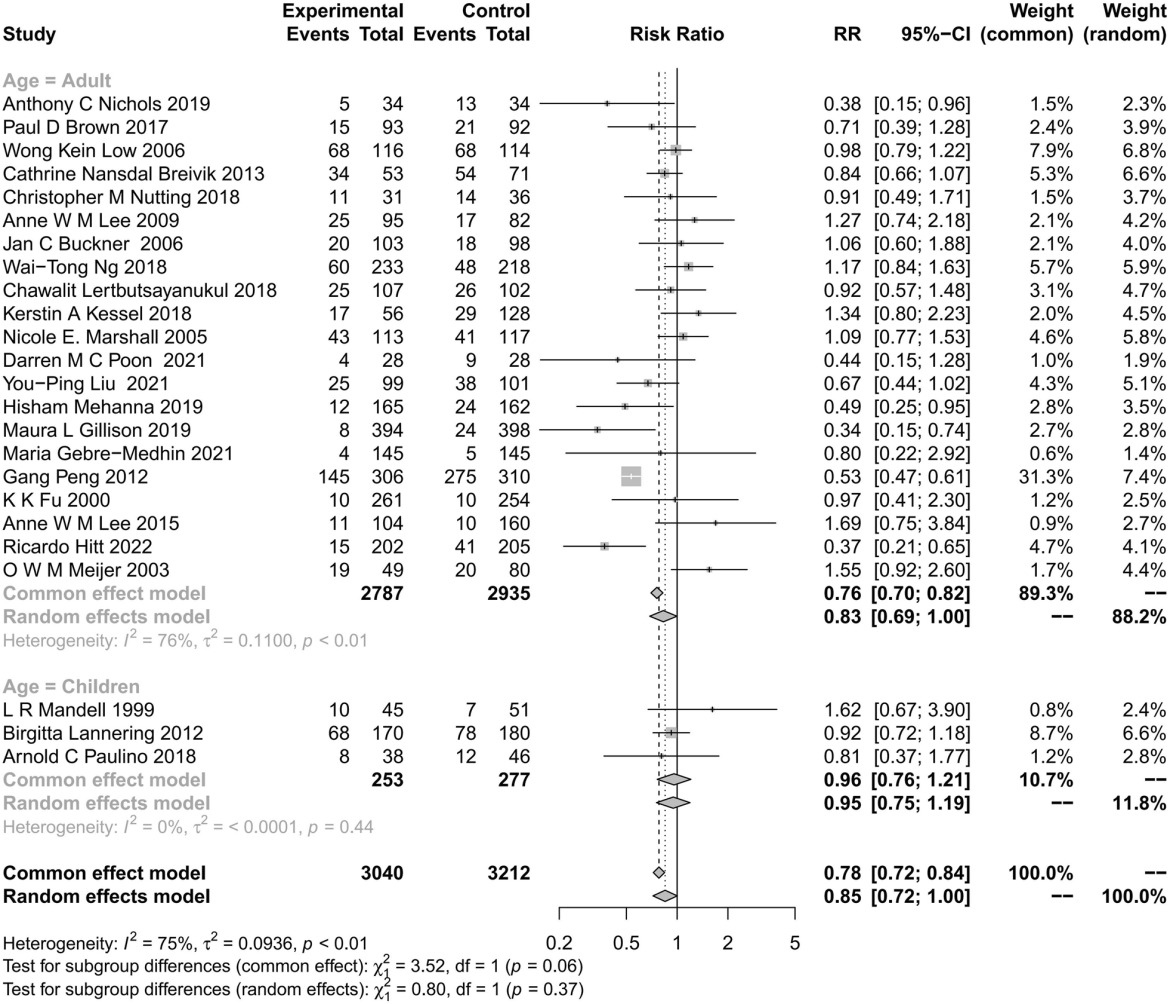
Subgroup Analysis of the Association of RT with hearing impairment by age

In the summary analysis of hearing impairment by comparing age groups, three studies included children under 18 years of age, and the combined effect value of hearing impairment was 0.95 (95% CI, 0.75–1.19; P = 0.44; I^2^ = 0%) (Fig. 4). Twenty-five studies reported hearing loss in adults, with the combined value being 0.83 (95% CI, 0.69–1.00; P < 0.01; I^2^ = 76%). The probability of hearing toxicity in children is higher than in adults.

##### Subgroup analysis of the association of RT with hearing impairment by tumor type

**Fig. 5:**
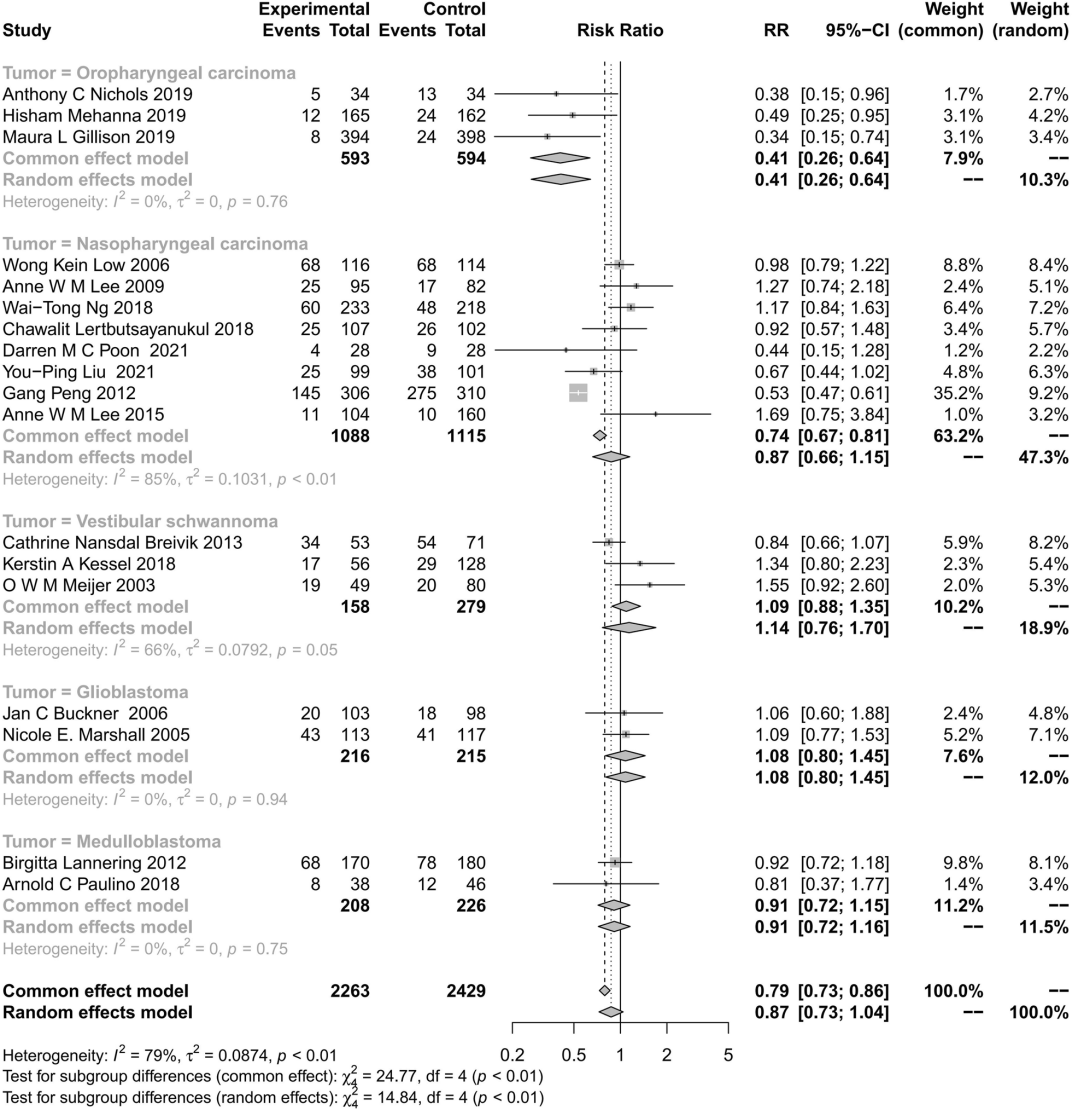
Subgroup Analysis of the Association of RT with hearing impairment by Tumor type

Oropharyngeal carcinoma appeared to have the lowest hearing impairment associated with radiotherapy (OR, 0.41, 95% CI, 0.26–0.64; P = 0.76; I^2^ = 0%). The hearing impairment of glioblastoma patients after irradiation was also relatively evident (OR, 1.08, 95% CI, 0.80–1.45; P = 0.94; I2 = 0%). Radiotherapy-associated hearing impairment in vestibular adenomas was high among all primary tumors included in the literature (OR, 1.14, 95% CI, 0.76–1.70; P = 0.05; I^2^ = 66%) (Fig. 5).

##### Subgroup analysis of the association of RT with hearing impairment by mean cochlear radiation dose

One randomized controlled study covered experimental versus control arms and involved different average cochlear doses. Therefore, when calculating cochlear dose-related radiotherapy hearing impairment, it was divided into each arm and its corresponding dose. A total of 887 individuals reported mean cochlear radiation dose and hearing loss in 10 arms. The mean cochlear radiation dose of the 3 arms was in the range of 30-40 Gy, with the probability of total hearing impairment being 27% (95% CI, 0.19–0.35; P = 0.42; I^2^ = 0%). When the cochlear radiation dose was 40-50 Gy, the combined value of total hearing impairment was 28% (95% CI, 0.19–0.39; P < 0.01; I^2^ = 85%). When the cochlear radiation dose increased to 50-60 Gy, the probability of total hearing impairment was the highest, at 35% (95% CI, 0.26–0.44) (Additional file 1: Fig. S2).

### Publication bias and sensitivity analysis

The funnel plots of hearing impairment included in the study were roughly symmetrical (Additional file 1: Fig. S3). The Egger test was also conducted to assess whether there was publication bias in this study. No significantly different results emerged, with p = 0.126 for Egger’s test. The combined effect value of the sensitivity analysis was 0.85 (95% CI, 0.72–1.00), indicating that the results were stable (Additional file 1: Fig. S4).

## Discussion

The study encompassed 28 randomized controlled trials, involving 6,252 patients, to evaluate ototoxic effects in cancer patients after radiotherapy. Factors such as mean cochlear irradiation dose, primary tumor, radiotherapy modality (or technique), and patient age may influence the risk of hearing impairment. IMRT radiotherapy-associated ototoxicity was less common than conventional radiotherapy. Stereotactic radiotherapy appeared to be a better option for hearing protection than radiosurgery. Children are at a higher risk of hearing impairment than adults. Over half of patients with vestibular neuroadenoma experience hearing impairment after radiation therapy. The average cochlear radiation dose is strongly associated with hearing impairment, and the radiation dose to the cochlea must be precisely controlled. To the best of our knowledge, this is the first comprehensive meta-analysis to analyze ototoxic injury caused by radiotherapy.

Previous literature has discussed the relationship between ototoxicity and radiotherapy. Theunissen and others conducted a study on sensorineural hearing loss (SNHL) caused by radiotherapy of head and neck tumors, suggesting that factors influencing the risk of SNHL included cochlear radiation dose, population age, and follow-up time [40]. Radiation-related ototoxicity involving auditory structures is multifactorial in nature. Radiation affecting the external auditory canal may lead to increased soft tissue susceptibility to infection and may necessitate regular removal of the cochlea to keep it dry [41]. Sensorineural hearing loss typically occurs at doses greater than 30 Gy [42, 43]. The risk of ototoxicity increases in patients receiving combined treatments, such as radiotherapy and platinum chemotherapy [44, 45].

Discussions about the optimal treatment strategy for techniques, prescription dosing, and segmentation are based on the need to prioritize curing the tumor while maintaining an acceptable risk of complications. Pediatric brain and head and neck malignancies requiring dose escalation, as well as adult skull base malignancies, are internationally recognized indications for proton therapy, exhibiting good local control, survival, and acceptable toxicity rates [46, 47]. Due to the lack of robust prediction models of photons and protons for this toxicity, it is impossible to predict the risk of hearing loss based on the patient's disease and treatment characteristics. The Normal Tissue Complication Probability (NTCP) model has been employed in previous studies to guide clinical judgment of proton beam therapy (PBT) [48]. In S. Gaito et al.’s study, the risk reduction of secondary tumors with PBT was estimated to be considerable compared with conventional photon radiotherapy through modeling studies. The clinical benefit of PBT primarily depends on the location of the tumor relative to the organ at risk and on the prescribed dose [48]. A multicenter study evaluating proton therapy and volumetric modulated arc therapy (VMAT) by establishing an NTCP model demonstrated that the reduction of NTCP in the population had a significant impact on auditory toxicity (VMAT: 8.0%; Proton: 3.3%). A significant reduction in the median population was observed in the proton-radiotherapy program, which provided auditory complications as well as a reduced risk of secondary brain cancer [49]. Previous research has also shown that proton therapy can effectively lower the dose of normal tissue surrounding patients with low-grade glioma (LGG). Compared with proton therapy, IMRT poses a two-fold higher risk of secondary intracranial tumors [50]. Fortin et al. conducted photon intensity modulation and proton radiotherapy in 50 children. Using proton and photon RT dose distributions, the intelligence quotient (IQ) and hearing loss probability of each ear were estimated by a Monte Carlo model. They concluded that compared with photon RT, proton RT is expected to reduce the adverse effects of RT on IQ and hearing [51].

Early radiation-induced ototoxicity is associated with mucosal edema, inflammation, and scaling of the outer, middle, and inner ear tissues [52]. The outer ear, middle ear, and inner ear may be affected, but otitis media is more common. It is related to middle ear effusion and can cause hearing loss, earache, and otorrhea, which usually subside after a few weeks [53]. The most prevalent toxicity is sensorineural hearing impairment. Arterial microvascular fibrosis and obliterative endarteritis often occur in the blood vessels of the inner ear's spirochetes, leading to degeneration and atrophy of the smooth muscle of the inner ear and the outer hair cells of the cochlea [54]. These hair cells are located at the base of the cochlea and are more sensitive to ionizing radiation than internal hair cells. They are responsible for hearing high frequencies, so high frequencies are more susceptible than low frequencies [55].

In this meta-analysis, the influence of age, radiotherapy mode, primary tumor, and mean cochlear dose on ototoxic hearing impairment is discussed. It can be concluded that the dose delivered to the inner ear (more precisely, the cochlea), radiotherapy technique (three-dimensional conformation), tumor type, and the age of the radiotherapy population are closely related to radiation-induced ototoxicity. The primary sites of radiation-induced ototoxicity are the paranasal sinuses, nasal cavity, nasopharynx, and parotid glands [56]. In the case of large tumors, the risk is undoubtedly greater. The ototoxicity of radiotherapy alone is related to the total dose received by the cochlea. Most authors in this study selected a threshold dose between 30 and 60 Gy. The probability of total hearing loss between 30 and 40 Gy was 27% (95% CI: 0.19–0.35), while the probability of total ototoxicity between 50 and 60 Gy was 35% (95% CI: 0.26–0.44). Charlotte et al. performed pure-tone audiometry at 0.250–16 kHz before and after treatment in 101 patients with head and neck cancer treated with modulated radiotherapy. They indicated that high frequencies might be affected early [57]. Keilty et al. assessed hearing in 340 children receiving radiotherapy. Mean cochlear dose, time after radiotherapy, cisplatin dose, and carboplatin dose were associated with increased assessment grades of hearing loss. If the mean cochlear dose is > 4 Gy, the cumulative incidence of high-frequency hearing impairment (> 5 kHz) at 50 years after radiotherapy is greater than 30%. Children who are treated with RT, especially those also receiving chemotherapy, are at a higher risk of hearing impairment and should have lower cochlear restraint [58]. They recommend an average cochlear dose of ≤ 30 Gy as a target for reducing Hodgkin's lymphoma risk [58]. To understand ototoxicity in radiotherapy patients, it is important to collect results reported by the clinician at baseline and during follow-up, in addition to tests such as audiograms, as this may partially overcome the inadequacy of hearing testing [59].

In this study, the subgroup analysis of hearing impairment with different radiotherapy techniques was analyzed. From the results, it is evident that the impact of carbon ions on hearing loss is minimal, making it a more reliable choice. Before the advent of IMRT, traditional two-dimensional radiation therapy was used. The response to radiation therapy depends on the sensitivity of tumor cells to radiation. In theory, higher radiation sensitivity leads to better therapeutic effects. However, the damage caused by treatment to the surrounding normal tissues also increases. Heavy particles, such as carbon ions, release energy to centrally explode tumor cells, maximizing the effect of radiotherapy and reducing damage to surrounding healthy tissues. Huang et al. divided 26 pediatric patients receiving medulloblastoma treatment into two groups, receiving conventional radiotherapy or IMRT, respectively. The pure tone audiogram was detected, and hearing function was graded from 0 to 4 according to the toxicity standard of the pediatric oncology group. They concluded that, compared to the traditional RT group, the IMRT group had a lower average decibel hearing threshold at each frequency. The overall incidence of ototoxicity in the IMRT group was low. Thirteen percent of the IMRT group had grade 3 or 4 hearing loss, compared with 64% of the conventional RT group [60]. In the study by Erner et al., 54 patients with low-grade and middle-grade chondrosarcoma of the skull base received carbon ion radiotherapy. After a median follow-up period of 33 months, only one tumor patient had sensory hearing loss in the inner ear. The patient retained useful hearing and did not use hearing aids [61].

The first-line treatment for radiation ototoxicity is drugs, which can be injected into the ear with a vasoconstrictor [52]. If this treatment is not effective, tympanoplasty may be required to treat middle ear effusion. Bone conduction hearing aids can be provided if symptoms associated with damage to the outer or middle ear persist, such as when mucosa is irreversibly damaged. They allow bypassing of the outer and middle ear, directly stimulating the sensory cells of the cochlea. Hyperbaric oxygen therapy can also be used as a treatment option for most subjects. Xu et al. discussed the treatment of 96 nasopharyngeal carcinoma patients with effusion otitis media after radiotherapy. They were divided into 3 groups: simple auricular point plus aspiration, tympanic membrane fenestration plus cauterization, tympanic membrane fenestration plus tympanic membrane tube insertion. Finally, they concluded that intensive local care after eardrum insertion can effectively reduce the incidence of ear complications after radiotherapy [62].

## Limitations

This study had several limitations. First, there are few RCTs providing average doses of cochlea, making it difficult to analyze the link between doses and ototoxicity on a large scale. Second, potential bias may exist due to differences in tumor stage and underlying disease in the included population. Third, the number of RCTs involved in each radiotherapy technique is small.

## Conclusions

In this study, randomized controlled trials were analyzed to compare the association of various factors with radiotherapy-related ototoxicity. Radiation design patterns and doses, population characteristics, and tumor characteristics are all intricately linked to ototoxicity. Children treated with RT, particularly those receiving chemotherapy, have a higher risk of hearing impairment; therefore, their cochlear restraint should be lower. To gain a better understanding of hearing toxicity, it is crucial to collect clinician-reported results at baseline and during follow-up, in addition to tests such as audiograms.

## Supplementary Information

**Additional file 1**. **Fig.S1.** Bias risk assessment chart. **Fig.S2.** Subgroup Analysis of the Association of RT With Hearing Loss by Cochlear irradiation dose. **Fig.S3. **Funnel chart of total hearing loss. **Fig.S4.** Sensitivity analysis of hearing loss.

## Data Availability

The authors confirm that the data supporting the findings of this study are available within the article.
