# Supplementary material for: The relevance of ototoxicity induced by radiotherapy
Source: Radiat Oncol. 2023 Jun 3;18:95. doi: 10.1186/s13014-023-02268-7 (PMC10239118; doi:10.1186/s13014-023-02268-7)

Fig.S1. Bias risk assessment chart


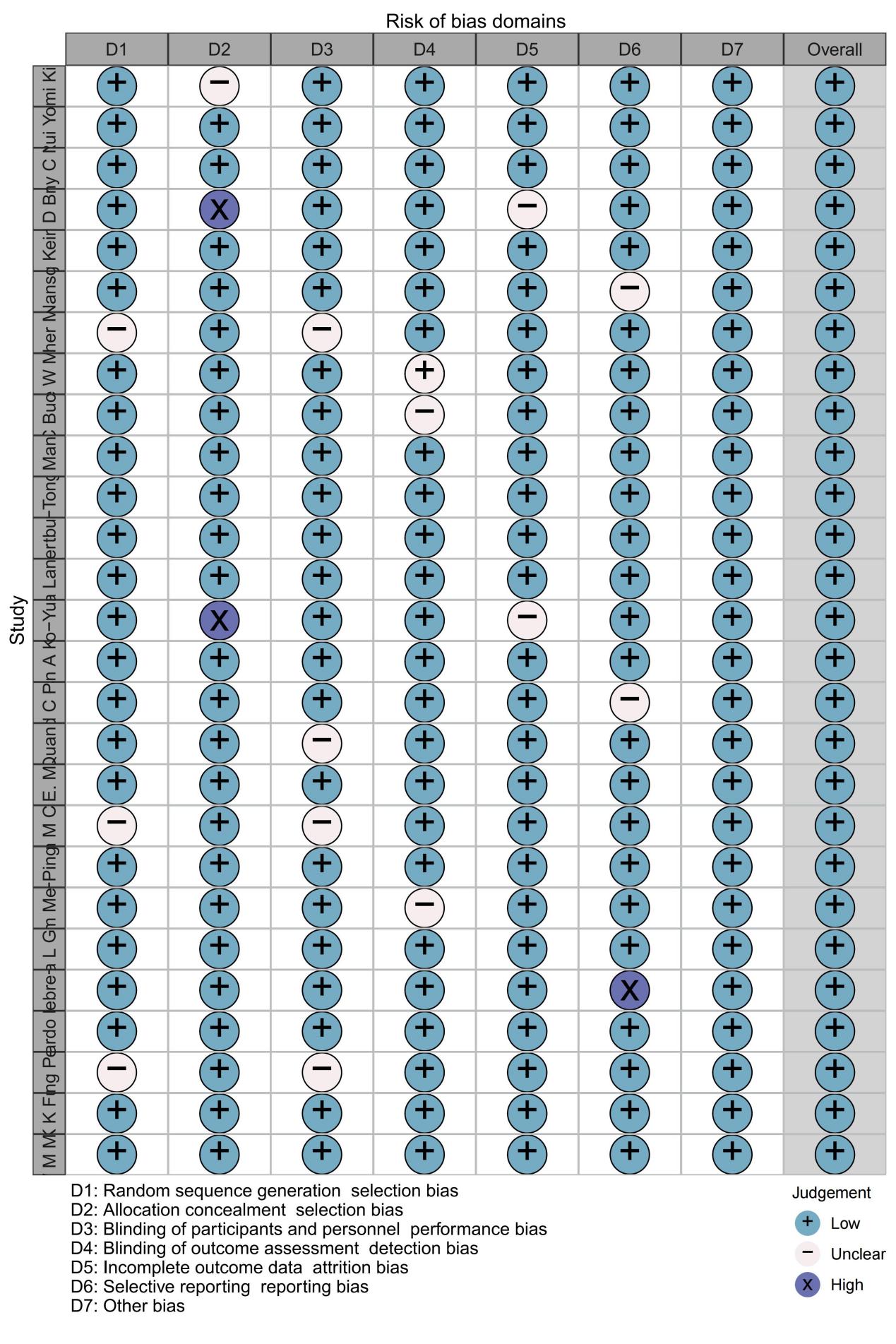


Fig.S2. Subgroup Analysis of the Association of RT With Hearing Loss by Cochlear irradiation dose


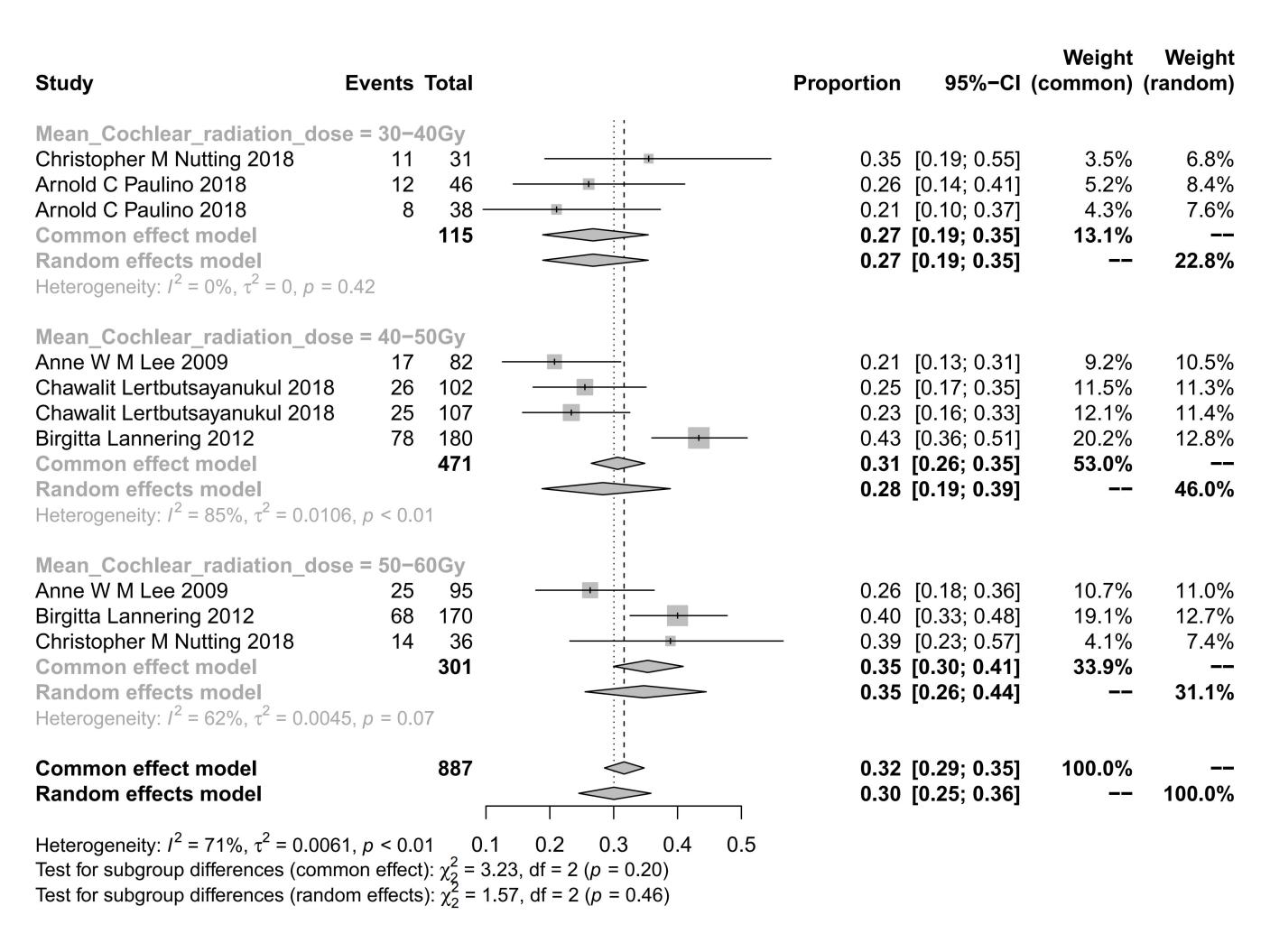


Fig.S3.Funnel chart of total hearing loss


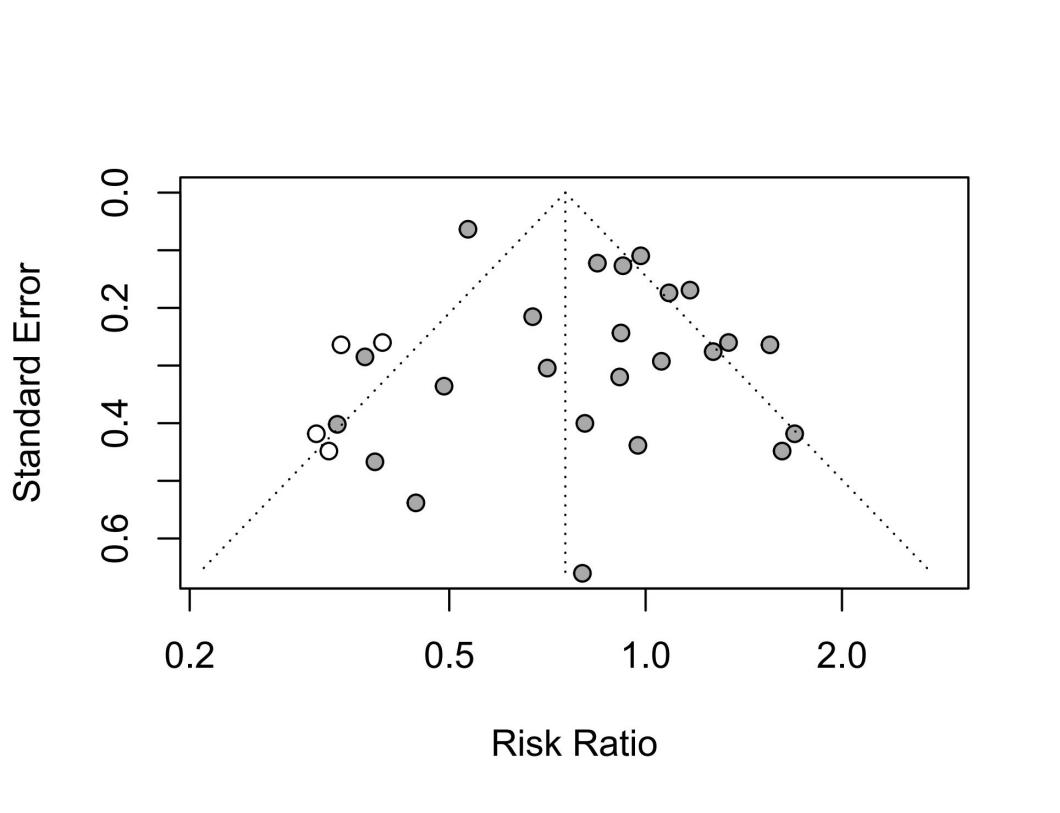


Fig.S4.Sensitivity analysis of hearing loss


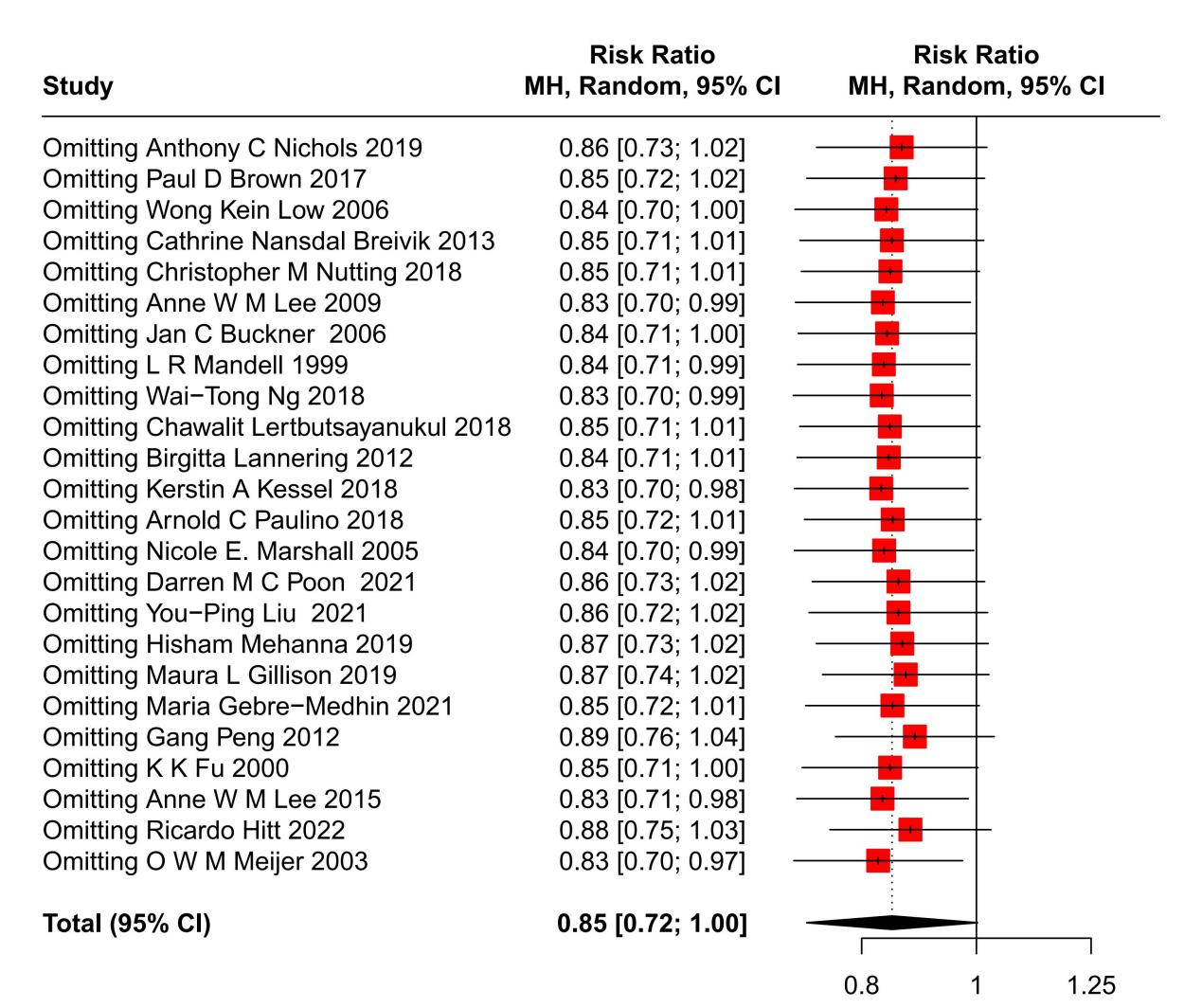

Supplement: Supplementary file 1 — Additional file 1. Fig.S1. Bias risk assessment chart. Fig.S2. Subgroup Analysis of the Association of RT With Hearing Loss by Cochlear irradiation dose. Fig.S3. Funnel chart of total hearing loss. Fig.S4. Sensitivity analysis of hearing loss. [file 13014_2023_2268_MOESM1_ESM.docx]
